# Superheated Dry Air

**Published:** 1899-02-11

**Authors:** 


### "SUPERHEATED DRY AIR."

Clinical experience has fully proved the utility of hot dry air in the treatment of stiff joints and of many painful rheumatic, gouty, and other conditions which interfere with mobility. Already there are many forms of apparatus in the market by which heat may be applied one way or another to various parts of the body; and, indeed, we seem threatened with what may almost be called a "boom" in hot air. We do not at the present moment propose to hold the balance between these competitors for medical favour, but we think it is time to point out the desirability of exactness of scientific language in discussing the question. First, then, as to the curious term, "superheated dry air"; surely this is a redundancy. "Superheated" is a term applied to steam, and means that it is heated to such a degree, apart from contact with water, that it acts as a gas, and is thus able to take up more water. In this sense—namely, as meaning that it is capable of evaporating water—dry air is always superheated whatever its temperature; and if the word can properly be applied to a gas at all, which we doubt, it would be far more appropriate to speak of the therapeutic employment of hot superheated air than of "superheated dry air." But the word is unnecessary, and its use implies inexactitude of thought; hot dry air ought to be sufficiently descriptive. The term is, however, creeping into medical literature, and it is time that its use was restricted to cases in which it has a proper meaning.

Then it would be well if medical men would bear in mind the great difference between the application of radiant heat and that of hot air. It is just possible, though at present nothing is really known about it, that the radiations from intensely hot sources may have some peculiar virtue, but, however this may be, it is certain that it is quite as necessary with radiant heat as with hot air that the air around the limb should be dry, and, in order that it may keep dry, that it should circulate. And this brings us to the last point, namely, that, although these very hot air baths are so effectual, they are effectual not by means of making the limb to which they are applied hot, but by virtue of the action set up in the limb to keep itself cool. This is mere commonplace to those who know, but it may be worth stating. Great as is the stress laid upon the fact that the temperature in the baths used runs up to 400 deg. Fahr., no one, of course, ever felt such a temperature. Any nerve would be killed and well cooked before it reached 170 deg. It is the protective actions set up to resist the very, very much lower temperatures, which alone reach the living tissues, that are the effective agents. Three things, at least, happen, perhaps many more about which we know nothing; but three things we know happen—perspiration takes place, and by its evaporation prevents this great heat reaching the body at all; a very greatly increased circulation in the part takes place, by which such of the heat as does get in is carried away and distributed throughout the rest of the body; and a considerably increased heat-loss, involving a proportionally increased circulatory activity, takes place in the body at large. No doubt, by dint of one or other of these disturbances of the tissues much morbid stuff is removed, just as the fire brigade in putting out a conflagration also swills the streets. But, as in the latter case, it is the water not the fire which makes the pavement clean, so it is the resistance to the heat and not the heat itself that cures the stiffness. All of which is but elementary, although it seems sometimes to be forgotten by some who advocate these special forms of treatment.

